# Six years of a national antimicrobial stewardship programme in Scotland: where are we now?

**DOI:** 10.1186/s13756-015-0068-1

**Published:** 2015-06-29

**Authors:** Clare Colligan, Jacqueline Sneddon, Gwen Bayne, William Malcolm, Gill Walker, Dilip Nathwani, on behalf of the Scottish Antimicrobial Prescribing Group

**Affiliations:** Scottish Antimicrobial Prescribing Group, Scottish Medicines Consortium, Healthcare Improvement Scotland, Delta House, 50 West Nile Street, Glasgow, G1 2NP UK; HAI and Infection Control Group, Health Protection Scotland, NHS National Services Scotland, 4th Floor, Meridian Court, 5 Cadogan Street, Glasgow, G2 6QE UK; NHS Education for Scotland, Westport,102 Westport, Edinburgh, EH3 9DN UK; NHS Tayside, Ninewells Hospital & Medical School, Ward 42, East Block, Dundee, DD19SY UK

**Keywords:** Antimicrobial stewardship, Structural and function indicators, Antimicrobial resistance

## Abstract

**Background:**

The Scottish Antimicrobial Prescribing Group (SAPG) was established in 2008 to lead delivery of the national antimicrobial stewardship programme. We performed a national self-reported survey in 2014 to evaluate stewardship activities delivered by regional Antimicrobial Management Teams (AMTs). An on-line survey was developed utilising validated indicators from a published European study along with questions specific to the Scottish context. Descriptive statistics were used to evaluate the responses received.

**Findings:**

The survey was completed by 14 of the 15 AMTs (response rate 93 %). Results demonstrated good compliance with 9 of the 10 key European indicators included in the survey; 7 (50 %) of AMTs achieved all 9 indicators and 14 (100 %) of AMTs achieved at least 6 out of 9 indicators (67 %). Progress was also demonstrated across a range of stewardship activities and areas for further work were identified.

**Conclusions:**

The survey results suggest the national stewardship programme in Scotland has reached maturity but consolidation and ongoing development are required. Collaborative working between SAPG and AMTs together with central funding has been key to achieving this level of success.

## Introduction

Antimicrobial resistance is recognised as a major global threat to healthcare and a post-antibiotic era is a real possibility [1, 2]. Antimicrobial stewardship programmes are vital to contain resistance and preserve the efficacy of the antimicrobial armamentarium particularly for multi-drug resistant gram negative infections emerging in the UK and elsewhere [3, 4]. The 2013 UK Antimicrobial Resistance Strategy places stewardship high in its recommendations [5] and is complemented by the 2014 refresh of the Scottish Management of Antimicrobial Resistance Action Plan (ScotMARAP) [6]. Healthcare in Scotland is delivered via one national National Health Service (NHS) board providing elective surgery and 14 regional NHS boards (11 mainland and 3 island boards), which provide both hospital and community based care. The Scottish Antimicrobial Prescribing Group (SAPG) was established in 2008 to lead and co-ordinate delivery of the original ScotMARAP and includes representatives from all mainland boards. SAPG have supported multi-professional Antimicrobial Management Teams (AMTs), one per board as sub-groups of the Drug and Therapeutics Committee, to implement stewardship across primary and secondary care. These AMTs include, as a minimum, a lead clinician, a microbiologist and an antimicrobial pharmacist but most also include an Infection Control and a primary care representative plus other clinicians. With the publication of these new strategy documents [5, 6], SAPG agreed it was timely to review the structure and function of AMTs, identify ongoing challenges and compare progress across Scotland. Measurement of stewardship and the need for common structure and process indicators for hospital stewardship programmes was recommended in the recent transatlantic taskforce on antimicrobial resistance (TATFAR) report [7]. Buyle et al [8] developed and validated a series of structure indicators using a multidisciplinary panel from four European countries who identified a series of 58 indicators, ranked them using a scoring system and then agreed the value of each via a face-to-face consensus meeting. We have used 9 of Buyle’s 10 key indicators adapted to the Scottish context along with questions specific to delivery of ScotMARAP and other SAPG initiatives to evaluate our progress.

## Method

A survey was developed utilising a Survey Monkey^©^ on-line tool with four domains of questions exploring various aspects of stewardship. The tool was tested by two boards to identify any practical issues or ambiguity in the questions. A web link to the survey was then sent via email to all 15 AMT Lead Clinicians, including those in the two test boards, who were asked to discuss with AMT colleagues and submit a response on behalf of the team.

## Results

### Survey participation

An excellent response was obtained with all boards completing the survey except one small island board serving less than 0.5 % of the Scottish population.

### Comparison with European validated indicators

**Fig. 1:**
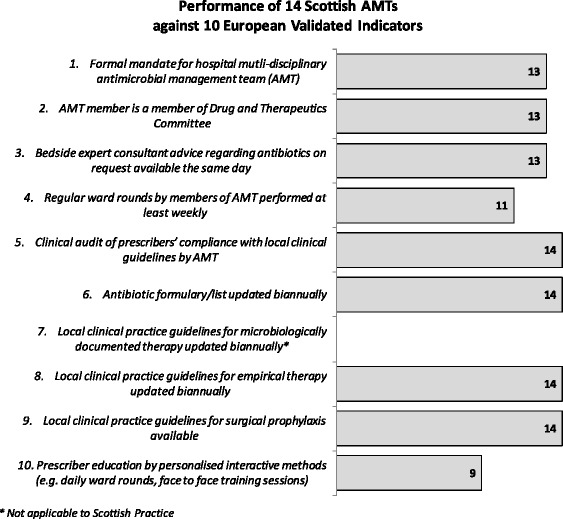
Performance of 14 Scottish AMTs against 10 European validated Indicators

Self-reported performance against these indicators is shown in Fig. 1. All boards are achieving at least six of the key European indicators and seven boards (50 %) are achieving all nine.

### Additional indicators across the 4 domains

**Fig. 2:**
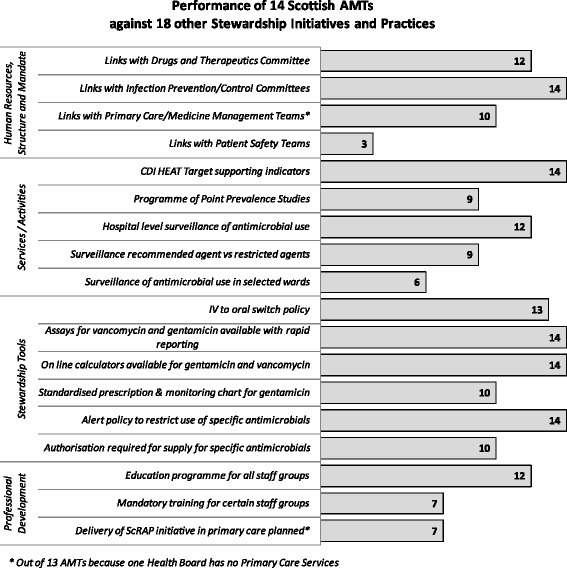
Performance of 14 Scottish AMTs against 18 other Stewardship Initiatives and Practices

A selection of the indicators is shown in Fig. 2 and highlights include:

- High levels of ‘excellent’ or ‘good’ collaborative working with Drug and Therapeutics Committees, Infection Prevention/Control Teams and Primary Care Teams. Engagement with Patient Safety Teams less effective.
- Good engagement with audit activities and surveillance of antimicrobial use at hospital level in most boards. Surveillance at ward level was less widespread due to local computer system capability and staff resource.
- High usage of a variety of stewardship tools to support safe and appropriate use of antimicrobials.
- A formal education programme on stewardship available in most boards but training mandatory in only half of the boards and mostly for Junior Medical Staff. Only half of boards delivering the primary care resource aimed at reducing unnecessary antibiotic use.

## Discussion

Charani et al [9] suggested that a whole system approach is essential to the success of stewardship and our survey response rate indicates good engagement with stewardship activities across Scotland. A recognised flaw in our approach is that responses were self-reported and therefore not validated. The achievements assessed using validated structure quality indicators [7] together with specific Scottish measures suggest a mature stewardship programme. The dedicated central funding [10] provided to support the SAPG infrastructure and antimicrobial pharmacists in every board has allowed local AMTs to meet the recommendations within the original ScotMARAP document. There is, however, no room for complacency and we must consolidate current success and develop new approaches.

A collaborative and integrated approach to stewardship, infection prevention and patient safety has been widely commended [11] and has been a key focus in Scotland to support the Healthcare Associated Infection agenda. Therefore, it is reassuring to confirm this joined up approach together with Drug and Therapeutics Committee links which ensures local strategic and operational direction and accountability. Each board has its own local antimicrobial policies which are developed and reviewed by the AMT and formally approved by the local Drug and Therapeutics Committee. The relationship with patient safety teams, involved in improving sepsis care and preventing infection, requires further development as only 3 boards reported ‘excellent’ or ‘good’ collaborative working and 4 boards reported it to be ‘minimal’. However early examples of success, within individual hospitals, are emerging and could be the basis of spread to other areas.

Surveillance of antimicrobial use is a key stewardship activity and boards are engaged with these activities to varying levels. Antimicrobial data is available through national systems; the Prescribing Information System (PIS) provides data for primary care prescribing at GP Practice level while the Hospital Medicines Utilisation Database (HMUD) provides information on antimicrobial use at hospital level only. Data analysis at ward level is variable and dependant on individual boards’ information systems as well as staff resources for data extraction. The proposed introduction of electronic prescribing across Scotland will facilitate ward level surveillance.

Restricting specific broad spectrum antibiotics such as the carbapenems has been adopted by all boards in combination with formal authorisation by an infection specialist in many to support our carbapenem reduction strategy [12].

In 2009 in response to increasing rates of *Clostridium difficile* infection (CDI) across Scotland national scrutiny measures were introduced involving a target for all boards to reduce CDI rates. To support this target a key priority has been restriction of antimicrobials most associated with CDI within local policies [13] and boards were mandated to collect and feed back clinical audit data to confirm this restriction in the form of prescribing indicators for hospital and primary care [14]. To provide assurance of compliance with restrictive policies this data is also collated at national level utilising a web-based system and results are reviewed quarterly by SAPG. In addition to this mandatory audit of prescribing, a programme of point prevalence surveys are carried out in acute hospitals in most boards to evaluate the quality of prescribing and results are fed back to clinical and managerial groups. Some boards also report auditing in clinical areas if sub-optimal prescribing has been identified or if surveillance data highlights increased use of restricted antibiotics.

An important but challenging element of stewardship is minimising harm from antimicrobial therapy both in terms of reducing resistance and also adverse effects at individual patient level. Restrictions on the use of antimicrobials with a high risk of CDI have resulted in increased use of gentamicin with potential for increased rates of renal toxicity and ototoxicity. Therefore SAPG commissioned a patient safety initiative including development of on-line dosage calculators for both gentamicin and vancomycin [15]. These are now the principal method of calculating initial and subsequent doses of these antibiotics and a national standardised gentamicin prescribing and monitoring chart [15] has also been adopted by most boards to guide safe practice.

No stewardship initiative would be complete without an educational component. There has been significant investment in development of national education resources to support antimicrobial stewardship [16]. These are well utilised by all boards to provide face-to-face and e-learning and most boards have a formal education programme which is mandatory for specific staff groups in half of these boards. To support a national target for reduction of antibiotic use in primary care an education resource was launched in 2013 for use within GP practices [17]. Although only half of boards have committed to deliver it within 2014–15 the remainder plan to do so in 2015–16 and SAPG will commence a formal evaluation in 2015.

## Conclusion

A national co-ordinated response led by SAPG and embraced by local AMTs has resulted in NHS Scotland being well positioned to take forward the challenges laid out in ScotMARAP2 [6] and the UK AMR strategy [5]. National engagement has been confirmed by this survey showing that key validated indicators of antimicrobial stewardship have been well established. Continued prioritisation and investment will allow us top consolidate this success and further utilise national and local data to drive improvement [18]. We believe that the use of pragmatic indicators commended by TATFAR, that can be locally adapted, are a valuable means of providing assurance about the function of stewardship programmes at a national and local level. We hope our survey data outlines their value and will support the implementation of these indicators across other healthcare settings.
